# Zirconium-Modified Medium-Entropy Alloy (TiVNb)_85_Cr_15_ for Hydrogen Storage

**DOI:** 10.3390/ma17081732

**Published:** 2024-04-10

**Authors:** Karel Saksl, Miloš Matvija, Martin Fujda, Beáta Ballóková, Dagmara Varcholová, Jakub Kubaško, Jens Möllmer, Marcus Lange, Mária Podobová

**Affiliations:** 1 Faculty of Materials, Metallurgy and Recycling, Technical University of Košice, Letna 9, 042 00 Kosice, Slovakia; milos.matvija@tuke.sk (M.M.); martin.fujda@tuke.sk (M.F.); dvarcholova@saske.sk (D.V.); jakub.kubasko@student.tuke.sk (J.K.); 2 Institute of Materials Research, Slovak Academy of Sciences, Watsonova 47, 040 01 Kosice, Slovakia; bballokova@saske.sk (B.B.); mpodobova@saske.sk (M.P.); 3 Institut für Nichtklassische Chemie e.V., Permoserstraße 15, 04318 Leipzig, Germany; moellmer@inc.uni-leipzig.de (J.M.); lange@inc.uni-leipzig.de (M.L.)

**Keywords:** medium-entropy alloy, (TiVNb)_85_Cr_15_, zirconium, hydrogen absorption, metal hydride

## Abstract

In this study, we investigate the effect of small amounts of zirconium alloying the medium-entropy alloy (TiVNb)_85_Cr_15_, a promising material for hydrogen storage. Alloys with 1, 4, and 7 at.% of Zr were prepared by arc melting and found to be multiphase, comprising at least three phases, indicating that Zr addition does not stabilize a single-phase solid solution. The dominant BCC phase (**HEA_1_**) is the primary hydrogen absorber, while the minor phases **HEA_2_** and **HEA_3_** play a crucial role in hydrogen absorption/desorption. Among the studied alloys, **Zr4** (TiVNb)_81_Cr_15_Zr_4_ shows the highest hydrogen storage capacity, ease of activation, and reversibly retrievable hydrogen. This alloy can absorb hydrogen at room temperature without additional processing, with a reversible capacity of up to 0.74 wt.%, corresponding to hydrogen-to-metal ratio H/M = 0.46. The study emphasizes the significant role of minor elemental additions in alloy properties, stressing the importance of tailored compositions for hydrogen storage applications. It suggests a direction for further research in metal hydride alloys for effective and safe hydrogen storage.

## 1. Introduction

The transition to a hydrogen and fuel cell-based economy presents a compelling opportunity for a greener energy sector, currently reliant on fossil fuels. Central to this transition is the development of efficient hydrogen storage methods, crucial for ensuring safety, effectiveness, and economic viability. Among these methods, solid-state storage has potential to store more hydrogen per unit volume compared to liquid or gas storage options. Since this approach has been introduced, significant attention has been devoted to exploring various types of metal hydrides as solid-state hydrogen storage materials [1,2]. Research in this area has primarily focused on enhancing the gravimetric and volumetric capacities of these materials, improving their thermodynamic and kinetic properties for hydrogen absorption/desorption, and ensuring long-term cycling stability [2,3,4,5,6,7,8].

Recently, new materials called medium- (MEAs) and high-entropy alloys (HEAs) are being explored for their potential to store hydrogen [3,9]. Such alloys were initially defined as those containing four or more principal elements with a concentration being between 5 at.% and 35 at.%. Later, another definition was also proposed, which suggests that the alloys can be classified as MEAs if their configurational entropies fall between 1 *R* and 1.5 *R* (*R* is the universal gas constant) and HEAs above 1.5 *R* [10,11,12]. Upon exposure to hydrogen, these alloys undergo a transformation into metal hydrides, known for their hydrogen storage capabilities. Since the properties of these hydrides depend heavily on their structure and composition, MEAs/HEAs, with their vast range of possible combinations, offer a great opportunity to design advanced hydrogen storage materials for clean energy storage and transportation.

The potential of medium-/high-entropy alloys (MEAs/HEAs) for hydrogen storage became particularly exciting when Sahlberg et al. [13] reported a high hydrogen-to-metal ratio H/M of 2.5 (equivalent to 2.7 wt.% H_2_) upon hydrogenation at 573 K and 5.3 MPa. However, further studies on the same HEA composition failed to replicate this high H/M ratio. For example, Ek et al. [14] measured capacities only around H/M = 2, but no results were shown where H/M of 2 was exceeded.

Two additional alloy systems stand out for their potential in room-temperature hydrogen storage: TiVNb-Zr (Ti_32.5_V_27.5_Nb_27.5_Zr_12.5_) and TiVNb-Cr ((TiVNb)_85_Cr_15_). Both exhibit a remarkable reversible capacity of H/M = 2, even after repeated cycling [15,16]. They absorb significant amounts of hydrogen (equivalent to 3.1–3.2 wt.% H_2_, or 2 H/M ratio) in less than a minute at room temperature and 0.2 MPa pressure. Increasing the temperature further enhances absorption speed, reducing the time needed to 20 s and the initial incubation period to just 35 s. The disadvantage of these alloys, however, is the relatively high temperature of hydrogen desorption from their volume. For example, for (TiVNb)_85_Cr_15_, the desorption temperature at a rate of 5 K/min starts at 150 °C, but to completely release hydrogen from the alloy, a temperature of 450 °C must be reached [17]. Hydrogen desorption from the Ti_32.5_V_27.5_Nb_27.5_Zr_12.5_ alloy occurs within a temperature range of 224 °C to 405 °C [18].

In this work, we investigated the effect of small additions of Zr (1, 4, and 7 at.%) on both the stability of solid solution formation and hydrogen absorption and desorption in (TiVNb)_85_Cr_15_. The effect of adding a small amount of Zr is nicely demonstrated by the study of Yang et al. on the alloy (VFe)_60_(TiCrCo)_40−x_Zr_x_ (0 ≤ x ≤ 2) [19]. Despite slightly reduced total storage capacity from 2.1 wt.% to 1.88 wt.%, the Zr element significantly improves its cyclic stability during hydrogen absorption/desorption cycles. Furthermore, it was observed that the zirconium addition caused also reduction in hydrogen desorption plateau pressure.

## 2. Materials and Methods

### 2.1. Material Design

In an effort to develop alloys with enhanced sorption properties, we employed the (TiVNb)_85_Cr_15_ precursor, to which we added a small amount of Zr (1, 4, and 7 at.%). The precursor itself forms a single-phase supersaturated solid solution with a BCC (body-centered cube) lattice [17]. We attempted to empirically predict whether the addition of Zr would preserve the single-phase structure applying Hume-Rothery’s rules for obtaining solid solution alloys [9]. In accordance with these principles, the creation of solid solutions is preferred in alloys where the elements share comparable atomic sizes, electronegativities, and valences, while also possessing identical crystalline structures [17,20]. In the empirical approach, factors representing discrepancies in atomic size (*δ*), concentration of valence electrons (*VEC*), the enthalpy of mixing (Δ*H_mix_*) and a parameter *Ω* that links the enthalpy of mixing, the entropy of mixing (Δ*S_mix_*), and melting temperature (*T_m_*) are determined according to the following equations [9,21]:

$$\delta =\sqrt{\sum c_{i}{\left(1-\frac{r_{i}}{\bar{r}}\right)}^{2}\;}\times 100$$

$$VEC=\sum c_{i}{\;VEC}_{i}$$

$$∆H_{mix}=\sum_{i<j}4{\;H}_{ij}{\;c}_{i}{\;c}_{j}$$

$$\Omega =\frac{T_{m}∆S_{mix}}{\left|\Delta H_{mix}\right|}$$

where $T_{m}=\sum_{i=1}^{n}c_{i}\;{\left(T_{m}\right)}_{i}$, and $∆S_{mix}=-R\sum c_{i}\ln c_{i}$. In the equations, *r_i_* and *VEC_i_* stand for the atomic radius and valence electron concentration of element *i*, respectively; $\bar{r}=\sum c_{i}r_{i}$ is the average of atomic radius; *c_i_* and *c_j_* are the atom fractions of elements *i* and *j*; *H_ij_* is the enthalpy of mixing of elements *i* and *j* at the equimolar concentration in regular binary solutions; (*T_m_*)*_i_* is the melting temperature of element *i*; and *R* is the universal gas constant [9,22].

Guo and his team defined the stability region of saturated solid solutions where the atomic size difference parameter *δ* < 6.6% and Δ*H_mix_* falls within the range of −11.6 to 3.2 kJ·mol^−1^ [21]. Furthermore, if *Ω* > 1, then the Gibbs free energy is determined by the mixing entropy Δ*S_mix_*, which stabilizes the solid solution. The values of thermodynamic parameters for all investigated alloys, as well as the precursor, are listed in Table 1.

**Figure 1:**
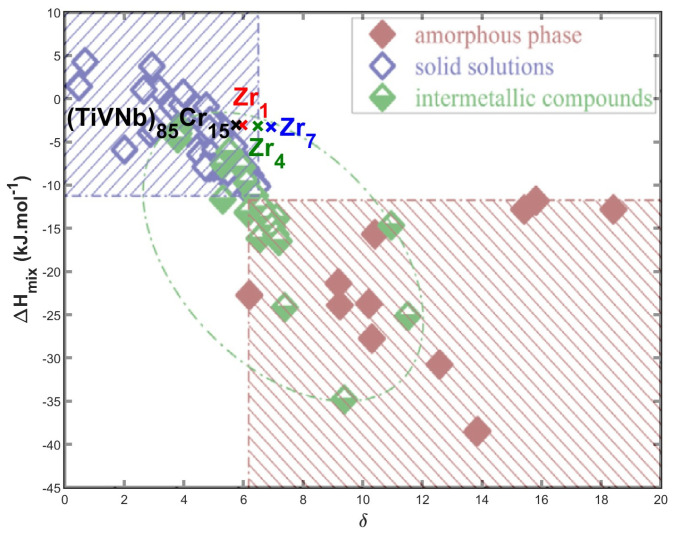
A *δ*—Δ*H_mix_* plot delineating phase selection in HEAs; dash dotted regions highlight individual region forming solid solutions, intermetallic compounds, and amorphous phase (graph adapted from [21]) with plotted parameters of the (TiVNb)_85_Cr_15_, **Zr1**, **Zr4**, and **Zr7** alloys.

The relationship between the *δ* and Δ*H_mix_* parameters can be depicted graphically, see Figure 1, where the blue shaded area represents the stability region of saturated solid solutions (MEAs/HEAs), the red shaded area represents the stability region of amorphous phases, and the region bounded by the green ellipse represents the region of intermetallic compounds. In this graph adapted from [21], we plotted parameters for both the precursor and the alloys under investigation containing a small addition of Zr.

From comparison of the thermodynamic parameters listed in Table 1 and Figure 1, the following conclusions can be drawn:

- All the alloys can be categorized as medium-entropy alloys since their mixing entropy values fall within the range of 1 R < Δ*S_mix_* < 1.5 R.
- High values of the parameter *Ω* were observed in all the alloys, suggesting their potential for forming a single disordered solid solution.
- Increasing Zr addition leads to a rise in the δ parameter (atomic size difference), causing the **Zr7** alloy ((TiVNb)_78_Cr_15_Zr_7_) to fall outside the empirically determined region of solid solutions.
- The value of the VEC parameter in all alloys is less than 6.87, indicating that the alloys should have a BCC structure [21].

### 2.2. Material Preparation

The alloys **Zr1** (TiVNb)_84_Cr_15_Zr_1_**, Zr4 **(TiVNb)_81_Cr_15_Zr_4,_ and **Zr7** (TiVNb)_78_Cr_15_Zr_7_ were synthesized by arc melting under an inert argon atmosphere, starting from pure elements purchased from Alfa-Aesar: Ti (99.99%), V (99.7%), Nb (99.8%), Cr (99%), and Zr (99.2%) in the Mini Arc Melting System MAM—1 furnace (Edmund Bühler GmbH, Bodelshausen, Germany). Initially, only Ti and Nb pieces were melted to form a binary alloy and subsequently, V, Cr, and Zr pieces were added and melted to obtain the desired alloy (this procedure was adopted to avoid incomplete dissolution of Nb pieces). The alloy was remelted five times, turning the piece upside down between each remelt step in order to improve their chemical homogeneity. The Ti, V, Nb, Cr, and Zr pieces used to synthesize the alloy have purity levels higher than 99.7%. Ti getter pieces were melted prior to the alloy’s synthesis to minimize oxygen content in the melting chamber.

### 2.3. Material Characterization

In the first step, the density of the alloys was determined by the Archimedes method, using the precise laboratory scales Kern ABT 120-4M (KERN & SOHN GmbH, Balingen, Germany) equipped with the special adapter ABT-A01 for density measurement. Metallographic cuts were prepared from the bulk alloys (buttons) using standard procedures, including mounting, planar grinding, rough polishing, final polishing, and etching. Microstructure and chemical composition of the prepared alloys were then obtained using the Jeol JSM 7000F (JEOL Ltd., Tokyo, Japan) scanning electron microscope equipped with an EDS detector. Microhardness tests HV0.3 were performed on the polished surface of the samples using a Wilson-Wolper Tukon 1102 hardness tester (Berg Engineering & Sales Company, Inc., Rolling Meadows, IL, USA) equipped with the Wicker type of microindenter. Ten indentations were made during the microhardness tests, and the mean value and standard deviation were calculated from the measurements. The nanoindentation hardness and elastic modulus were determined using the Nano Indenter G200 device manufactured by Agilent Technologies, Inc. (Chandler, AZ, USA). The measurement consisted of 30 indentation cycles, each applying a load of 50 mg for 15 s.

In the next step, the bulk alloys were pulverized using ball milling for 20 min in an argon atmosphere. The powder samples were then sieved in a glovebox to particles below 45 μm in size to maximize the active surface area for hydrogen absorption. An additional density measurement was performed on this powder fraction using the helium pycnometer AccuPyc II 1345 (Micromeritics, Norcross, GA, USA). Results from the Archimedean (bulk materials) and He pycnometry (powder) measurements were very similar, with differences only in the third decimal place. The phase composition analysis was conducted using the X-ray diffraction on the Philips X’Pert Pro diffractometer (Malvern Panalytical, Almelo, The Netherlands). The XRD measurements were performed in 2θ range from 20° to 120° with 0.03° step size and 25 s dwell time per step.

### 2.4. Hydrogen Absorption and Desorption Experiments

Hydrogen absorption measurements were conducted using the magnetic suspension balance (IsoSORP series, TA Instruments, New Castle, DE, USA), which can operate at pressures up to 50 MPa with a measurement accuracy of 0.05%. Each sample was measured according to the following protocol:

1. Approximately 0.5 g of powder alloy was placed into the reaction chamber of the magnetic suspension balance. The system was then sealed and evacuated to a rotary pump vacuum <0.02 bar (2 kPa).
2. The alloy was activated by exposure to low hydrogen pressure of approximately 0.1 MPa at room temperature for 1 h to reduce oxides on the surfaces of the powder particles. To remove the absorbed hydrogen, the sample was then heated to 400 °C for 3 h in a vacuum.
3. After activation, the reaction chamber was cooled to room temperature. Once reached, it was filled with hydrogen to a pressure of 2 MPa. The sample mass was monitored by the magnetic suspension balance. The chamber temperature (and thus that of the sample) was increased from room temperature to 250 °C in steps of 25 °C. At each step, the sample was held for 25 min. The aim of this isobaric measurement was to determine the temperature at which the sample starts significantly absorbing hydrogen.
4. After this measurement, the sample was cooled down to room temperature under 2 MPa of H_2_. Measurement in hydrogen after cooling allowed us to determine the amount of total absorbed hydrogen by the sample in step 3.
5. The chamber was then evacuated again and heated to 400 °C for 3 h to desorb hydrogen from the sample.
6. The chamber was heated to the temperature at which the alloy absorbed hydrogen significantly, and hydrogen was again introduced to the chamber at a pressure of 2 MPa. During this second absorption measurement lasting 1 h, the sample weight was monitored again.
7. Following hydrogenation, the sample was removed from the chamber for X-ray diffraction (XRD) and thermogravimetric analysis (TGA) using the Netsch Jupiter STA 449-F1 analyzer, Selb, Germany.

The hydrogen absorption raw data were further recalculated to amount of hydrogen absorbed per gram material using standard routines for buoyancy corrections [23].

## 3. Results and Discussion

The results of our material analysis on the Zr-modified (TiVNb)_85_Cr_15_ alloys are listed in the Table 2.

### 3.1. Chemical Composition and Density

EDX spectroscopy performed on the **Zr1**, **Zr4**, and **Zr7** bulk alloys (polished metallographic cross-sections) confirmed a chemical composition close to the nominal. The maximum deviation obtained was 2 at.%, which is typical for arc-melted alloys. The density of these alloys was ~6.56 g.cm^−3^, and due to only a slight change in the concentration, its value changed only at the second decimal place.

### 3.2. Phase Composition

**Figure 2:**
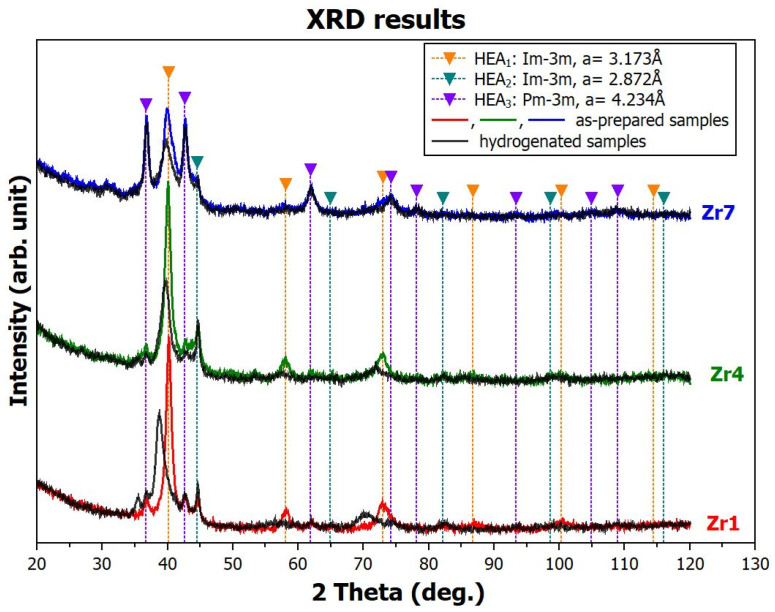
The XRD patterns of the as-prepared **Zr1**, **Zr4**, and **Zr7** samples are shown in color. The black curves correspond to these alloys after hydrogenation. The positions of the Bragg peaks of the identified phases are indicated by triangle symbols and vertical lines.

Phase analysis was performed on all samples in powder form, both in their initial state (as-prepared) and after hydrogenation. Figure 2 shows the XRD patterns of the as-prepared samples of **Zr1**, **Zr4**, and **Zr7** in red, green, and blue, respectively. The black patterns are from these alloys but after hydrogenation.

#### 3.2.1. Analysis of the As-Prepared Samples

Phase analysis of the as-prepared samples demonstrates that they are not single-phase, but rather consist of multiple phases. It is important to note here that detailed structural analysis of the (TiVNb)_85_Cr_15_ precursor revealed that even this alloy is not completely single-phase [24,25]. Samples **Zr1** and **Zr4** consist of a dominant BCC phase (Space Group (S.G): *Im-3m*) with a lattice parameter of a = 3.173 Å (in the figure, shown in orange and labeled as **HEA_1_**). In addition to this phase, at least two other minor phases are present: a second BCC phase (S.G: *Im-3m*) with a significantly smaller lattice parameter of a = 2.872 Å (shown in green and labeled **HEA_2_**) and a primitive cubic phase (S.G: *Pm-3m*) with a larger lattice parameter of a = 4.234 Å (purple, labeled **HEA_3_**). For this last phase, it cannot be ruled out that it is a C14 Laves phase (S.G: *P6_3_/mmc*) similar to that in the work of [26], but this cannot be confirmed based on the performed experiment. All these phases are present also in the **Zr7** sample, but their proportions are different. The **HEA_1_** and **HEA_3_** are approximately equally represented, while the amount of the **HEA_2_** decreased.

#### 3.2.2. Analysis of the Hydrogenated Samples

After hydrogenation, the samples **Zr4** and **Zr7** exhibit similar XRD patterns to those in the as-prepared state. The Bragg peaks from the **HEA_1_** have lower intensity, which is probably due to the refinement of the structure (reduction in crystallite size), and are slightly shifted to the left, which means that the lattice parameter of the original phase has slightly increased due to the presence of hydrogen. The pattern from the **Zr1** sample shows a larger change with a significant increase in the lattice parameter of the **HEA_1_** phase. Interestingly, the peaks from **HEA_2_** and **HEA_3_** remain unchanged in all patterns. These results lead us to the following conclusions:

- Based on the analysis of the Bragg peak shift (lattice parameter changes), we believe that the **HEA_1_** phase is the phase that significantly absorbs hydrogen in all samples. The **HEA_2_** and **HEA_3_** phases are practically unaffected by hydrogen, which means that they either do not absorb hydrogen or release it after removal from the reaction vessel. Unfortunately, this study did not allow us to perform in situ XRD experiments during hydrogenation of our alloys. Synchrotron sources would be the most suitable for this purpose. However, we are not aware of any beamlines that allow experiments at hydrogen pressure of 20 bar.
- The increase in the lattice parameter of the **Zr1** sample is a manifestation of the chemical bonding of absorbed hydrogen in the metal matrix. As will be shown later, samples **Zr4** and **Zr7** absorb hydrogen equally and even more, but they bind it with weaker bonds, which causes hydrogen to escape from the matrix at ambient conditions.
- Since no permanent changes in the diffraction profiles were induced by hydrogen, it can be concluded that hydrogen is dissolved within interstitial positions in the absorbing phases and does not form hydrides with a completely different crystallography.

### 3.3. Microstructures

**Figure 3:**
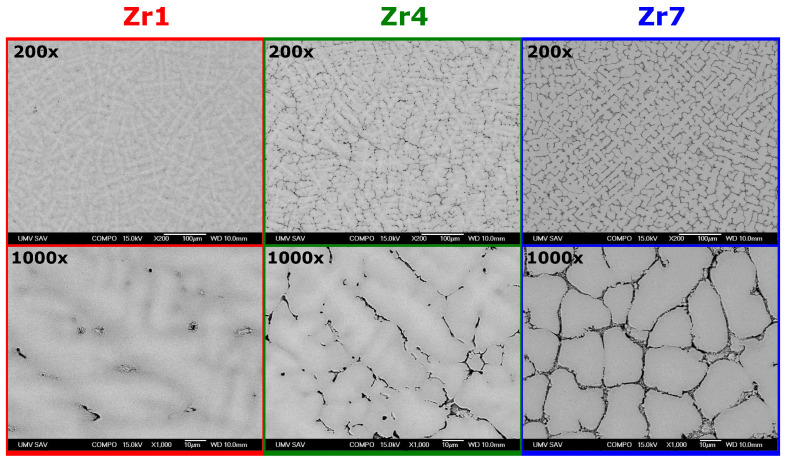
The SEM BSE images of microstructures of the Zr-modified (TiVNb)_85_Cr_15_ alloys. The top row shows a lower magnification (200×) while the bottom row shows a higher magnification (1000×).

Microstructures of the Zr-modified (TiVNb)_85_Cr_15_ alloys are shown in Figure 3. The images were obtained by SEM operated in the backscattered electron (BSE) mode. In all alloys, a primary dendritic heterogeneous microstructure can be observed, which documents the presence of multiple chemically distinct phases. In the case of the **Zr7** alloy, a higher proportion of secondary **HEA_3_** phase can be observed visible at boundaries of the **HEA_1_** grains. This observation is consistent with our XRD results. As mentioned earlier, the **HEA_3_** can be the C14 Laves phase, which crystallizes eutectically at the boundaries of the **HEA_1_** grains.

### 3.4. Hydrogen Absorption and Desorption

#### 3.4.1. Absorption

**Figure 4:**
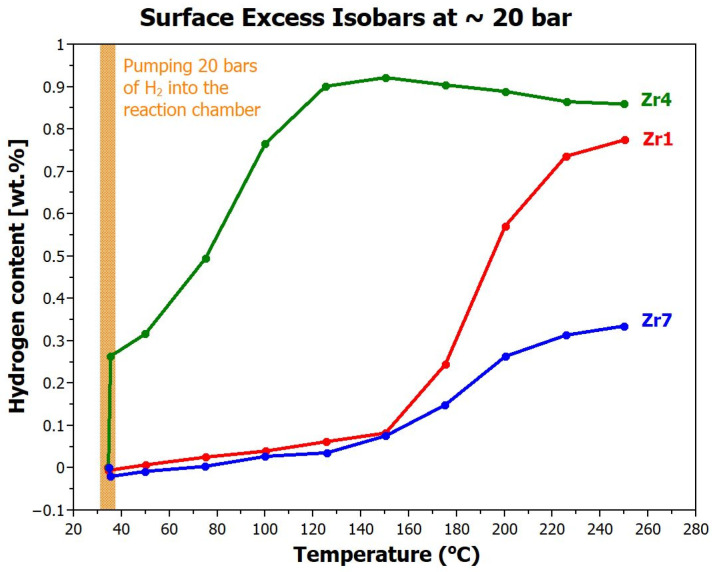
Amount of hydrogen stored in the **Zr1**, **Zr4**, and **Zr7** alloys (in wt.%) under constant pressure and increased temperature (isobars).

Hydrogen absorption in the Zr-modified (TiVNb)_85_Cr_15_ alloys was evaluated by weighing the samples at elevated temperatures under hydrogen gas environment of constant pressure of 2 MPa. This pressure was chosen because most commercial low-pressure metal hydride storage tanks operate at this pressure. The alloys were heated under isobaric conditions in the temperature range from RT to 250 °C in steps of 25 °C, with each step lasting for 25 min. Figure 4 illustrates absorption capabilities of the **Zr1**, ** Zr4,** and **Zr7** alloys under constant pressure and increasing temperature.

By comparing these isobaric curves, the following observations can be made:

- The alloy with the lowest zirconium content, **Zr1**, begins to absorb hydrogen at temperatures above 150 °C. The maximum storage capacity of 0.77 wt.% (corresponding to H/M = 0.47) was reached at the highest measurement temperature of 250 °C.
- The **Zr4** alloy is activated already at room temperature. A significant increase in this alloy weight was observed already when the reaction chamber was filled with hydrogen. The maximum amount of hydrogen absorbed by this alloy is 0.92 wt.% (H/M = 0.57). As can be seen, from 150 °C, the alloy starts to desorb hydrogen, so it is very likely that if we increased the pressure of gaseous hydrogen in the chamber, its absorption capacity would be higher.
- By adding additional 3 at.% Zr to the alloy (sample **Zr7**), this trend was significantly reverted. Activation again occurs only at high temperatures above 150 °C. The overall hydrogen absorption capacity is low, only 0.34 wt.% (H/M = 0.21), and only at the highest measured temperature.

#### 3.4.2. Desorption

**Figure 5:**
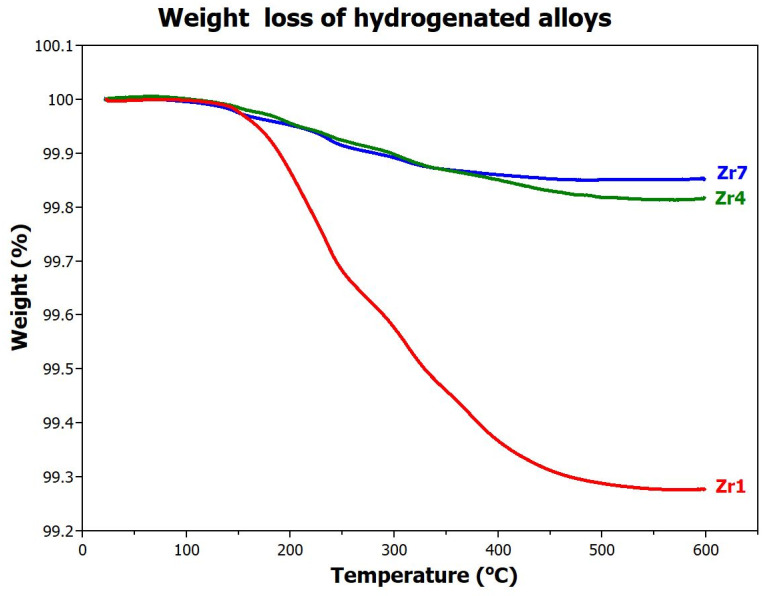
TG measurements of the **Zr1**, **Zr4**, and **Zr7** samples at a heating rate of 10 K/min.

As mentioned in the previous chapter, hydrogen desorption from the **Zr4** alloy also occurs at 2 MPa of H_2_ at temperatures above 150 °C. To determine parameters of complete hydrogen desorption, the samples were first fully hydrogenated (see Section 2.4), then removed from the chamber, and subsequently subjected to thermogravimetric (TG) experiments. During the TG measurement, weight loss of the sample was determined as a function of increasing temperature at a heating rate of 10 K/min in argon atmosphere. Figure 5 shows the desorption curves of all alloys.

Based on these results, the following conclusions can be formulated:

- The **Zr1** sample contains a large amount of chemically bounded hydrogen, which significantly starts to desorb from the alloy at ~160 °C. Complete release of hydrogen from the metal lattice occurs only at 500 °C. These results approximately correspond to the desorption temperatures from reference [25]. The amount of hydrogen released in this way is 0.73 wt.%. For this alloy, only 0.04 wt.% of hydrogen is, therefore, available for reversible low-temperature (up to 160 °C) absorption/desorption.
- The situation is completely different for the **Zr4** alloy where the amount of hydrogen released at 600 °C is significantly lower, only 0.18 wt.%. This means that this alloy has up to 0.74 wt.% (H/M = 0.46) hydrogen available for reversible use.
- The situation is similar for the **Zr7** alloy, but since this alloy absorbs the least, the amount of reversibly available hydrogen is only 0.19 wt.%.

This study clearly demonstrates the significant impact that a small addition of a suitably chosen element can have on hydrogen absorption and desorption properties.

## 4. Conclusions

In this work, we focused on the influence of adding a small amount of Zr to the medium-entropy alloy (TiVNb)_85_Cr_15_, which is currently considered one of the most promising materials for practical hydrogen storage. The alloys we prepared with additions of 1, 4, and 7 at.% Zr are multiphase, consisting of at least three phases. Therefore, adding Zr does not allow stabilizing a single-phase supersaturated solid solution. The major BCC phase (referred to as **HEA_1_** in the article) is the primary hydrogen-absorbing phase, while **HEA_2_** and **HEA_3_** show minimal hydrogen uptake. However, the presence of these two minor phases is crucial for hydrogen absorption and desorption from these materials.

In terms of the amount of hydrogen stored, ease of activation, and amount of reversibly retrievable hydrogen, the **Zr4** (TiVNb)_81_Cr_15_Zr_4_ is the best of all the alloys we studied. This alloy, which can be prepared easily by arc melting and powdered by crushing, can absorb hydrogen at room temperature without any additional treatment. The amount of reversibly usable hydrogen in low-pressure tanks is up to 0.74 wt.%, which corresponds to H/M = 0.46. We can, therefore, conclude that precipitating the right amount of **HEA_2_** (around 10 vol%) and **HEA_3_** (around 3 vol%) phases activates the main **HEA_1_** phase, allowing it to absorb more hydrogen. Moreover, it is important to note the following: This alloy exhibits the lowest modulus of elasticity among all the studied alloys. In our latest unpublished work, we demonstrate the correlation between hydrogen absorption and the alloy’s modulus of elasticity. Moreover, this alloy does not contain rare earth elements, which China banned from exporting at the end of the year in 2023 [27].

The most commonly used alloy for hydrogen storage today is LaNi_5_, which can store approximately 1.2 wt.% hydrogen (H/M~1) at a pressure of 24 bar. It reaches full saturation at this pressure and room temperature within 7.5 min. For our **Zr4** alloy under the same conditions, this is approximately 0.27 wt.% (H/M = 0.17). This alloy, therefore, is not yet perfect from the application point of view, but this work points the way for future materials research on metal hydride materials and demonstrates how to activate MEAs/HEAs for hydrogen absorption.

Overall, the study highlights the significant impact of minor elemental additions on the hydrogen absorption and desorption properties of alloys. This underscores the importance of carefully selecting alloy compositions to tailor their performance for specific applications in hydrogen storage technologies. This article thus identifies one of the directions for further development and research of metal hydride alloys for safe hydrogen storage.

## Figures and Tables

**Table 1:** Thermodynamic parameters of the alloy (TiVNb)_85_Cr_15_ and the alloys with Zr additions investigated.

Alloy	δ [%]	Δ*H_mix_* [kJ·mol^−1^]	*Ω*	Δ*S_mix_* [J.K^−1^.mol^−1^]	*T_m_* [K]	*VEC*
**Precursor** (TiVNb)_85_Cr_15_	5.76	−3.04	8.37	1.36 *R*	2257	4.87
**Zr1** (TiVNb)_84_Cr_15_Zr_1_	5.96	−3.07	8.54	1.40 *R*	2255	4.86
**Zr4 **(TiVNb)_81_Cr_15_Zr_4_	6.48	−3.17	8.70	1.47 *R*	2251	4.84
**Zr7** (TiVNb)_78_Cr_15_Zr_7_	6.93	−3.27	8.69	1.52 *R*	2246	4.82

**Table 2:** Results from the measurement of Zr-modified (TiVNb)_85_Cr_15_ alloys. Chemical composition of the alloys determined by the EDX spectroscopy, density, microhardness HV0.3, elastic modulus, activation temperature of hydrogen absorption at pressure of 20 bar, maximum achieved storage capacity of hydrogen in the alloy, and the amount of residual hydrogen firmly chemically bounded to the alloy matrix.

Alloy *EDX Composition* [at.%]	Density [g.cm^−3^]	Hardness HV03	Elastic Modulus [GPa]	Activation Temperature [°C]	Maximum H_2_ Capacity [wt.%] *(H/M)*	Residual H_2_ Content [wt.%]
**Zr1** (TiVNb)_84_Cr_15_Zr_1_ *Ti*_28_ *V*_27_ *Nb*_30_ *Cr*_14_ *Zr*_1_	6.53	482 ± 8	130 ± 1	>150	**0.77** *(0.47)*	0.73
**Zr4 **(TiVNb)_81_Cr_15_Zr_4_ *Ti*_27_*V*_26_*Nb*_29_*Cr*_14_*Zr*_4_	6.58	463 ± 13	112 ± 4	<RT	**0.92** *(0.57)*	0.18
**Zr7** (TiVNb)_78_Cr_15_Zr_7_ *Ti*_26_*V*_25_*Nb*_28_*Cr*_14_*Zr*_7_	6.57	475 ± 17	136 ± 6	>150	**0.34** *(0.21)*	0.15

## Data Availability

Data are contained within the article.
